# Direct-to-Consumer Erectile Dysfunction Medications: is the Convenience Worth the Cost?

**DOI:** 10.1007/s11934-025-01302-3

**Published:** 2025-10-30

**Authors:** Michelle K. Li, Darshan P. Patel, Tung-Chin Hsieh

**Affiliations:** Department of Urology, University of California, San Diego, CA USA

**Keywords:** Cost, Direct to consumer advertising, Erectile dysfunction, Sexual health, Male, Humans

## Abstract

**Purpose of the Review:**

Erectile dysfunction (ED) is a highly prevalent disease, affecting over 24% of men in the United States. Factors such as privacy and accessibility often act as barriers to care for patients. With the emergence of telemedicine, direct-to-consumer (DTC) platforms have become popular avenues for patients to seek care. These platforms are especially attractive to patients with ED due to their promises of providing discrete and convenient care. Phosphodiesterase type 5 (PDE5) inhibitors remain the most commonly prescribed therapy for ED. This review evaluates recent literature over the past five years to better understand the economics of PDE5 inhibitors offered through DTC platforms.

**Recent Findings:**

Studies have shown that while DTC companies offer convenience, these medications are offered at a significantly marked up price compared to more conventional means, such as an in-person clinical visit and brick and mortar pharmacies.

**Summary:**

While DTC platforms offer a promising model for improving access to ED care, their economic trade-offs and clinical limitations must be considered. Ongoing research and transparent pricing are needed to ensure that all patients with ED can make informed choices that best meet their clinical and economic needs.

## Introduction

Erectile dysfunction (ED) is a highly prevalent disease, affecting over 24% of men in the United States [1]. Nonetheless, factors such as embarrassment, lack of awareness, accessibility, and privacy are often cited as barriers to care, leading to underdiagnosis and undertreatment [2]. The advent of direct-to-consumer (DTC) marketing platforms have become an attractive avenue for patients seeking ED treatment, due to these companies’ promises of privacy and decrease stigma [3].

Since 1980s, pharmaceutical companies have been utilizing direct-to-consumer (DTC) marketing platforms to promote highly profitable, branded treatment to patients [3]. The emergence of telemedicine – further accelerated by the COVID-19 pandemic – has fueled the growth of DTC platforms, with an estimated market size of $422.2 million by 2030 [4]. These platforms have become increasing popular to patients seeking urologic care [3]. Dietrich et al. identified a total of 147 DTC men’s health clinic that offered ED treatment, ranging from medications to platelet rich plasma and penile shockwave therapy [5]. However, the rapid growth of these services also raises important questions about the quality and cost of care that patients receive outside traditional clinical settings.

The purpose of this review is to provide a comprehensive analysis of recent literature examining the economics of ED medications offered through DTC platforms.

## Methods

A literature search was performed on PubMed and Google Scholar for journal articles published between the period of June 2020 and June 2025 using the keywords “erectile dysfunction medications”, “cost”, and “direct-to-consumer”. We specifically evaluated cost analyses for phosphodiesterase type 5 (PDE5) inhibitors. A total of three articles were identified that compared the cost of PDE5 inhibitors from DTC platforms to those from other sources.

## Current Literature

 Phosphodiesterase type 5 (PDE5) inhibitors remain one of the most widely used ED medications and is strongly recommended by the 2018 American Urological Association Guidelines for ED [6]. A 2020 study found that the United States market sold over 400 million dollars of sildenafil in a single quarter, with the drug accounting for 47% of the total revenue for ED medications [7]. 

 Brink et al. identified a total of 15 DTC platforms that provided consultation and prescription of PDE5 inhibitors through a strictly online platform [8]. Of the platforms, 10 offered free consultations, but these companies did charge for monthly or quarterly fees for prescriptions [8]. The PDE5 inhibitors sildenafil and tadalafil, were available for purchase on all platforms [8]. The minimum prices for sildenafil were noted to be $0.50 to $35 per pill, while those of tadalafil were noted to be $0.50 to $9.80 per pill [8]. This wide price range reflects not only the differences between generic and branded products but also the impact of subscription models, bundled telehealth services, and varying business strategies among DTC providers.

 Schenider et al. compared the costs of PDE5 inhibitors obtained via in-person physician visits and local prescription, Canadian online pharmacies that provide shipment to the United States, and DTC companies [9]. They specifically evaluated hims® and Roman®, the two largest DTC platforms [9]. The study elucidated that for a 90-dose prescription of various PDE5 inhibitors, DTC platforms had the highest mean and lowest price compared to local prescriptions. The Canadian pharmacy was also evaluated as a secondary outcome with mixed results in terms of price ranking. For instance, for sildenafil 20 mg, the mean local prescription cost is $191.51 and low cost is $125.45; the mean Canadian pharmacy cost is $240.25 and low cost is $169.34; and the mean DTC market cost is $227.50 and low cost is $195.00 [9]. However, these differences between these mean prices were not statistically significant [9]. The study did find that difference in cost of tadalafil 5 mg between DTC companies and local prescription and DTC companies and Canadian pharmacies were statistically significant (p < 0.01) [9]. For tadalafil 5mg, the mean local prescription cost is$205.79 and low cost is $125.80; the mean Canadian pharmacy cost is$207.59 and low cost is $195.34; and the mean DTC market cost is$727.50 and low cost is $720.00 [9].

 These findings are consistent with another study that demonstrated significant markup in PDE5 inhibitors on DTC platforms compared to local prescriptions [10]. This study included three DTC platforms: BlueChew®, hims®, and Roman®. The authors examined the prices for introductory packages of both sildenafil and tadalafil. The price per pill for sildenafil was $4.17, $6.50, $6.25, and $0.32, at BlueChew®, hims®, Romans®, and Cost Plus Drug Company, respectively [10]. Similarly, the prices were higher for tadalafil, which costed $0.36 per pill at Cost Plug Drug Company, but $6.25, $7.50, and $9.75, respectively at BlueChew®, hims®, and Romans® [10]. Cheaper options were available with commitment to longer term subscriptions, but the prices remained higher than from Cost Plus Drug Company. The differences in price were most pronounced at higher strength and quantities [10]. This study is limited in that they did not account for the cost of having a physician and the means to obtain a local prescription.

 Another important consideration is the lack of insurance coverage for many DTC prescriptions. Most DTC platforms operate on a cash-pay basis, and insurance is rarely accepted or processed for either the telehealth consultation or the medication itself. This can further increase the out-of-pocket cost for patients, especially when compared to local pharmacies that may accept insurance or offer generic medications at steep discounts through discount cards or pharmacy benefit programs. As a result, patients who choose DTC services for the sake of convenience may ultimately pay significantly more over time, particularly if they require ongoing therapy. However, it is important to note that many ED medications obtained through insurance may have a formulary or tier system. While most patients in our practice are able to obtain some coverage through their insurance, insurance may require prior authorization or be subject to exclusion policies. Furthermore, Medicare and Medicaid have a nationwide noncoverage for PDE5is as they are not deemed medically necessary. These limitations can make ED medications less accessible or even more costly than DTC platforms that can offer generic drugs without insurance restrictions.

 Nonetheless, while DTC companies offer a private and convenient platform for patients to obtain ED medications, current literature suggests that these benefits come with a considerable markup in price.

##  Discussion

 The evolving role of telemedicine has been central to the success of DTC platforms. The promise of privacy makes them particularly appealing to patients with urologic conditions, which often carry social stigma. While DTC companies can help reduce barriers to care for patients with ED by offering convenient and discreet services, this literature review demonstrates that these benefits frequently come with high monetary costs.

 From an economic perspective, the DTC model introduces both opportunities and challenges. On one hand, DTC platforms may offer cost savings related to reduced travel time, time off from work, and physician costs. On the other hand, as highlighted in recent studies, the actual price paid by patients for medications is often higher through DTC services than through traditional pharmacies or wholesale options. This is particularly evident for generic PDE5 inhibitors, which are widely available at a fraction of the cost in local pharmacies or through online wholesalers such as Cost Plus Drug Company. The markup observed on DTC platforms is frequently justified by the inclusion of telemedicine consultations and home delivery; however, for price-sensitive patients, these added conveniences may not outweigh the increased financial burden [11].

 Furthermore, studies have shown other considerable drawbacks beyond cost. One major concern is the inability to perform a comprehensive medical evaluation [12]. One of the Clinical Principles of the 2018 American Urological Association Guidelines for ED include counseling patients with ED that there may be concern for underlying cardiovascular disease [6]. The lack of in-person evaluation with DTC platforms can result in missed opportunities for diagnosis and ultimately, delayed medical intervention. Studies have shown that appropriate screening for cardiovascular disease is a cost-effective strategy to prevent atherosclerotic cardiovascular disease (ASCVD) events that can range from $8,000 to $36,000 over a 10-year period [13, 14]. It is crucial to consider these downstream expenditures in cost analyses as they are difficult to account for and often overlooked. Beyond the inability to screen for cardiovascular disease, DTC platforms are limited in their capacity to assess for other underlying causes of ED, such as hormonal imbalances, neurologic disorders, or psychological conditions. The absence of a physical examination and laboratory testing on DTC platforms may result in overlooked diagnoses and suboptimal outcomes for patients whose ED is secondary to another medical issue. Moreover, the lack of ongoing follow-up and patient-provider relationship inherent to DTC models further reduces opportunities for longitudinal care and adjustment of therapy as patient needs evolve. Another significant limitation of DTC companies is their narrow focus on oral PDE5 inhibitors. While these are often effective for many, they are not suitable for all patients. For patients who do not respond to, cannot tolerate, or have contraindications to PDE5 inhibitors, alternative treatments such as intracavernosal injection (ICI) therapy, vacuum erection devices, intraurethral suppositories, penile prostheses, and even psychological counseling should be considered [6]. DTC platforms rarely provide these options or the necessary education regarding their risks, benefits, and proper use. Furthermore, there is currently no literature comparing the cost-effectiveness of these alternative therapies provided on DTC platforms versus local pharmacies and clinic settings.

 Finally, while DTC platforms can reduce stigma and improve access for some, they may worsen disparities for others. Patients with limited digital literacy, unreliable internet access, or those who are not comfortable navigating online healthcare may be excluded. Additionally, the higher cost of medications on DTC platforms, as consistently demonstrated in the literature, may disproportionately impact those with limited financial resources, further widening the gap in access to effective ED care. Studies have shown that while Hispanics and African Americans are less likely to be exposed to DTC advertising, they are more likely to be influenced by it [15]. However, there are currently no studies that examine the demographics of DTC users for ED medications. Future research is needed to evaluate the racial and socioeconomic distribution of these individuals to identify possible health disparities.

 These findings extend beyond PDE5 inhibitors. Cost analyses have been performed on other urologic treatment such as testosterone replacement therapy [16, 17]. Technological barriers and inability to perform a physical exam have been again cited as limitations of DTC platforms [17]. While initial consultation with DTC platforms may be less costly than that with tertiary center through Medicare or private insurance, ongoing treatment was demonstrated to be significantly higher when obtained through DTC platforms [16]. For a 12-month period of intramuscular testosterone treatment, the price through the DTC platforms Hone, Regenex Health, and TRT Nation, ranged from $1,586 to$4,200, while the price through the tertiary center ranged from$134.01 to $1333.04 with different insurance models. These findings once again elucidate the premium cost of DTC platforms in exchange for convenience, privacy, and accessibility.

 One promising intervention for improving access and reducing costs of ED treatment is the reclassification of PDE5 inhibitors to over-the-counter (OTC) medications. Currently, PDE5 inhibitors are available only through prescription in most countries, which introduces barriers related to cost, embarrassment, and inconvenience for patients seeking treatment. Expert opinions from the Group Delphi consensus meeting in Germany have concluded the benefits of reclassifying PDE5 inhibitors to OTC drugs will far outweigh the risks [18]. These benefits include increased access, reduced stigma, and greater convenience. Furthermore, risk mitigation strategies such as clear labeling and patient information leaflets can be implemented. Transitioning PDE5 inhibitors to OTC would allow patients to purchase these medications directly from pharmacies or online platforms without a prescription, potentially lowering costs by eliminating the need for physician visits and prescription processing and reducing reliance on potentially unsafe or counterfeit sources. This approach aligns with global trends toward deregulation of certain prescription medications and could serve as a model for future cost analyses of DTC ED medication markets. However, similar to the DTC platform, the transition to OTC would pose concerns of bypassing appropriate cardiovascular screening and resulting in higher overall healthcare costs.

##  Conclusion

 Ultimately, DTC platforms have lowered barriers to care and normalized ED treatment for many men. However, the economic implications for patients are significant. For some, the value of privacy and convenience may justify the premium cost. For others, especially those with limited financial resources or complex medical needs, traditional care models may offer a more comprehensive and cost-effective solution. Ongoing research and transparent pricing will be essential to ensure that all patients with ED can make informed choices that best meet their clinical and economic needs. Research should also seek to elucidate health disparities that may result from these platforms which tend to be more expensive and require technological proficiency.

## Key References

- 8. Brink SM, Iarajuli T, Shin D. Characteristics of direct-to-consumer platforms offering erectile dysfunction treatment. Sex Med. 2023;11(4):qfad038. Published 2023 Aug 2. doi:10.1093/sexmed/qfad038. Evaluates the difference in costs of PDE5 inhibitors across different online DTC platforms.
- 9. Schneider D, Loeb CA, Brevik A, El-Khatib F, Jenkins LC, Yafi FA. Contemporary cost-analysis comparison of direct-to-consumer vs. traditional prescriptions of phosphodiesterase-5 inhibitors. Int J Impot Res. 2023;35(5):460–464. doi:10.1038/s41443-022-00567-3. Examines the differences in costs of PDE5 inhibitors from local pharmacies after a physician visit, online pharmacies, and DTC platforms.
- 10. Vercnocke J, Zhong A, Liaw A, Ginsburg K. The High Price of Anonymity: Comparison of Cost of Direct-to-Consumer Mail Order Pharmacies Compared With an Online Wholesale Pharmacy. J Urol. 2024;211(1):170–171. doi:10.1097/JU.0000000000003720. Evaluates the difference in cost of PDE5 inhibitors from DTC platforms compared to an online pharmacy.

## Data Availability

No datasets were generated or analysed during the current study.
